# Serum Metabolites as Potential Markers and Predictors of Depression-like Behavior and Effective Fluoxetine Treatment in Chronically Socially Isolated Rats

**DOI:** 10.3390/metabo14080405

**Published:** 2024-07-25

**Authors:** Dragana Filipović, Julica Inderhees, Alexandra Korda, Predrag Tadić, Markus Schwaninger, Dragoš Inta, Stefan Borgwardt

**Affiliations:** 1 Department of Molecular Biology and Endocrinology, “VINČA”, Institute of Nuclear Sciences—National Institute of the Republic of Serbia, University of Belgrade, 11000 Belgrade, Serbia; 2 Institute for Experimental and Clinical Pharmacology and Toxicology, Center of Brain, Behavior and Metabolism, University of Lübeck, 23562 Lübeck, Germany; julica.inderhees@uni-luebeck.de (J.I.); markus.schwaninger@uni-luebeck.de (M.S.); 3 Bioanalytic Core Facility, Center of Brain Behavior and Metabolism, University of Lübeck, 23562 Lübeck, Germany; 4 Department of Psychiatry and Psychotherapy, Center of Brain Behavior and Metabolism, University of Lübeck, 23562 Lübeck, Germany; alexandra.korda@uni-luebeck.de (A.K.); stefan.borgwardt@uksh.de (S.B.); 5 School of Electrical Engineering, University of Belgrade, 11000 Belgrade, Serbia; ptadic@etf.rs; 6 Department for Community Health, Faculty of Natural Sciences, Medicine, University of Fribourg, 1700 Fribourg, Switzerland; dragos.inta@unifr.ch; 7 Department of Biomedicine, University of Basel, 4001 Basel, Switzerland

**Keywords:** rat serum, depressive-like behavior, fluoxetine, metabolite profiling, machine learning

## Abstract

Metabolic perturbation has been associated with depression. An untargeted metabolomics approach using liquid chromatography-high resolution mass spectrometry was employed to detect and measure the rat serum metabolic changes following chronic social isolation (CSIS), an animal model of depression, and effective antidepressant fluoxetine (Flx) treatment. Univariate and multivariate statistics were used for metabolic data analysis and differentially expressed metabolites (DEMs) determination. Potential markers and predictive metabolites of CSIS-induced depressive-like behavior and Flx efficacy in CSIS were evaluated by the receiver operating characteristic (ROC) curve, and machine learning (ML) algorithms, such as support vector machine with linear kernel (SVM–LK) and random forest (RF). Upregulated choline following CSIS may represent a potential marker of depressive-like behavior. Succinate, stachydrine, guanidinoacetate, kynurenic acid, and 7-methylguanine were revealed as potential markers of effective Flx treatment in CSIS rats. RF yielded better accuracy than SVM–LK (98.50% vs. 85.70%, respectively) in predicting Flx efficacy in CSIS vs. CSIS, however, it performed almost identically in classifying CSIS vs. control (75.83% and 75%, respectively). Obtained DEMs combined with ROC curve and ML algorithms provide a research strategy for assessing potential markers or predictive metabolites for the designation or classification of stress-induced depressive phenotype and mode of drug action.

## 1. Introduction

Chronic psychosocial stress plays a significant role in developing major depressive disorder (MDD), also known as depression [1]. Among chronic psychosocial stressors, chronic social isolation (CSIS) is the most prevalent type of stress involved in developing symptoms of depression [2,3]. Given that research into the molecular mechanisms and treatments of depression is severely limited, the use of reliable animal models is crucial, one of which is the CSIS model [4,5]. This is a mild chronic stress that evokes various behaviors, neurochemical and neuroendocrine changes in adult rats, comparable to those seen in people suffering from depression. Importantly, symptoms are reversible with antidepressant treatments [5,6,7,8]. Recent studies showed alterations in the proteome and metabolome, and marker candidates in the prefrontal cortex (PFC) and hippocampus of CSIS rats and/or in antidepressant fluoxetine (Flx) effective treatment [9,10,11]. Moreover, changes in energy metabolism and mitochondrial dysfunction in both brain structures have been associated with depression [12,13].

The majority of antidepressants used today in clinical practice are based on the monoamine hypothesis of depression with a primary mechanism of blocking 5-hydroxytryptamine reuptake [14,15]. A commonly used drug to improve depressive symptoms in patients is Flx, as one of the most widely prescribed antidepressants [16], which also shows therapeutic efficacy in animals. Besides raising serotonin levels in the synaptic cleft [17,18], the antidepressant effect of Flx is associated with increasing neurogenesis, neural plasticity, and GABA signaling [19,20,21,22]. Although the effectiveness of Flx in depressive patients has undoubtedly been determined, its mode of action is not clearly understood. Therefore, intensive research is focused on more detailed insight into the molecular mechanisms of Flx action.

As part of this research, untargeted metabolomics is gaining importance. This approach has revealed metabolite changes in depression in both clinical [23,24,25] and animal studies [25,26,27], upon treatments [28,29]. Moreover, metabolite analysis can be useful in the search of potential biomarkers [30], addressing an important clinical need [31,32], to identify disease states and treatment effectiveness. Additionally, metabolite signatures can be further utilized for future sample classification through machine learning- (ML)-based data processing [33,34]. Hence, support vector machine (SVM) and random forest (RF) algorithms have been used in the field of biomarker discovery [35,36]. In a previous study, our group successfully established a prefrontal cortical-based metabolic profiling approach in CSIS rats that showed depression-like behavior and effective Flx treatment in CSIS rats by liquid chromatography–high resolution mass spectrometry (LC–HRMS). Focus was on the PFC due to its involvement in the regulation of emotion, cognitive processes, motivation and sociability [37]. This metabolic profiling revealed myo-inositol as a marker candidate for depressive-like behavior and sedoheptulose 7-phosphate, hypotaurine and acetyl-L-carnitine as potential markers for Flx efficacy in CSIS rats [9]. Moreover, these metabolic changes provide a basis for revealing molecular biomarkers of disease and targets for application treatment.

Moreover, relatively high sensitivity and accuracy, as well as the availability of access to a test sample, are necessary conditions for biomarkers to be used for disease diagnosis and treatment outcomes. Brain tissues represent suitable biological samples for studying depression [38]. However, brain biopsy samples from depressed patients are neither feasible nor convenient, while serum samples can be easily obtained without risk to the patient, making them a suitable option for clinical laboratories. Moreover, peripheral metabolic changes have been found in depression [39], indicating that metabolic changes associated with depressive etiology may generate detectable blood-based biosignatures that can be used for diagnosis and treatment response [40,41]. Thus, current research is focused on the samples of rat serum, as the preferred specimen for translational applications, which contain a broad spectrum of stable metabolites, which will generally reflect the body’s overall metabolic alterations brought on by CSIS and Flx treatment.

Hence, untargeted metabolic profiling of rat serum was performed by liquid chromatography–high resolution mass spectrometry (LC–HRMS) to identify and quantify the serum metabolic changes in CSIS (6-week) rats and/or following effective Flx treatment (lasting 3 weeks of 6-week CSIS). The forced swim test (FST) was used to distinguish rat phenotypes. The objective of this study was to identify circulating potential metabolite markers and predictors for differentiating or classifying CSIS-induced depressive-like behavior vs. control, and Flx efficacy in CSIS vs. CSIS, as well as obtaining a new insight into the molecular mechanisms of the stress-induced depressive-like behavior and Flx mechanism of action. In this study, univariate and multivariate analyses using *t*-test and partial least squares–discriminant analyses (PLS–DA), respectively, were applied to identify differentially expressed metabolites (DEMs) and test the separation of sample groups, respectively. The performance of each metabolite’s capacity as a potential marker for differentiating the CSIS-induced depressive-like behavior and Flx efficacy was assessed utilizing classical univariate receiver operating characteristic (ROC) curve analysis by the area under the curve (AUC). ML algorithms, including SVM with a linear kernel (SVM–LK) and RF [42,43], were applied to detect metabolites important in classifying CSIS vs. controls and Flx efficacy in CSIS vs. CSIS. To investigate if the intensity of serum metabolites may reflect the behavior despair in the CSIS model and Flx effectiveness, the relationship between all metabolites and immobility time in the FST was examined.

So far, no studies have examined serum-based metabolome profiling combined with ML algorithms to identify the potential capacity of markers and predictive metabolites to differentiate or classify CSIS vs. controls and Flx efficacy in CSIS vs. CSIS.

## 2. Methods

### 2.1. Animals

Adult male Wistar rats (2.5 months of age, 300–350 g body weight) were bred in the Animal Facility of VINČA Institute of Nuclear Sciences, National Institute of the Republic of Serbia, University of Belgrade. Rats were housed in groups of up to four per cage to a 12-hour light/dark cycle (light 0700–1900 h), in a temperature- (20 ± 2 °C), and humidity-controlled (55 ± 10%) environment, with free access to food (commercial rat pellets) and water ad libitum. Rats were monitored daily.

### 2.2. Fluoxetine–Hydrochloride Administration

The tablets of fluoxetine–hydrochloride (Flunisan 20 mg, Hemofarm, Vršac, Serbia) were crushed, dissolved by ultrasound in sterile MilliQ water, and then passed through Whatman No. 42 filter paper for filtration. The concentration of Flx in the water solution was measured using ultra-performance liquid chromatography [44]. Throughout the preparation process, a loss of 25% in Flx concentration was detected, which was taken into consideration during its administration in rats (15 mg/kg/day) [45]. A weekly body weight measurement was used to determine the appropriate dose of Flx (i.e., mg/kg). Rats given 15 mg/kg/day of Flx for 3 weeks had Flx concentration of 280 ± 50 ng/mL in Flx-treated controls and 203 ± 28 ng/mL in Flx-treated CSIS rats, determined 24 h following the last treatment [46]. A chosen dose of Flx produces serum levels in the rats that correspond to therapeutically effective concentrations in depressed patients receiving 20–80 mg/day of Flx (100–700 ng/mL) [47].

### 2.3. Experimental Design

**Figure 1:**
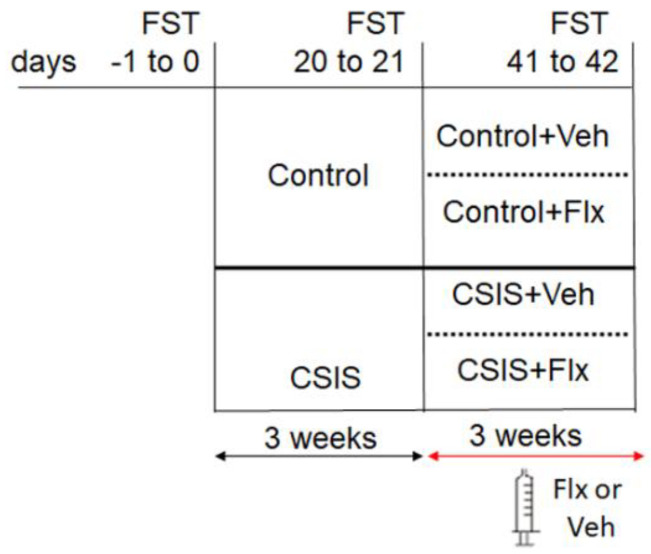
Experimental design of the study. CSIS—Chronic Social Isolation; Flx—fluoxetine; FST—forced swim test; Veh—vehicle (0.9% NaCl).

The graphical representation of the experimental design is presented in Figure 1. We employed a CSIS paradigm, as previously described [9]. Briefly, 50 rats were randomly divided at the beginning of the experiment (week 0) into two separate groups: CSIS (*n* = 30, housed individually, without tactile or visual contact), and control (*n* = 20, housed in groups of up to four). Rats were not subjected to any additional experimental treatments for the first 3 weeks. During the second 3-week period, intraperitoneal (i.p.) injections of Flx solution (15 mg/kg/day) were administered to half of each group of rats (Control + Flx and CSIS + Flx); the other rats received daily i.p. injections of physiological saline solution (1 mL) (Control + Veh and CSIS + Veh). Twenty-four hours following the behavior experiment, the rats were anesthetized with ketamine/xylazine solution (120/16 mg/kg) (Ketamidor^®^ 100 mg/mL, Richter Pharma AG, Wels, Austria/Xylazin Bio^®^ 20 mg/mL, Bioveta, Hané, Czech Republic), perfused with physiological saline, and sacrificed by decapitation from 9.00 to 12.00 h. Rats were classified into groups according to the results of the behavioral results using the FST.

### 2.4. Forced Swim Test

The FST was conducted to evaluate depressive-like and antidepressant-like behavior [48]. Rats were placed individually in a transparent cylinder Plexiglas apparatus (height 45 cm × diameter 28 cm) filled with 30 cm tap water at 24 ± 1 °C. At the onset of experiment FST was done in two sessions 24 h apart. The first session is the pre-test stage (15 min) and the second session is the test stage (5 min) [49]. During the 5-min period, immobility, swimming, and climbing time were recorded by Sony HDR-PJ410 camera. Two observers, who were blinded to the setting of the experiment, analyzed the data. The immobility time was used as a measure of behavioral despair [49]. The test was conducted before the onset of the experiment (week 0-baseline) and at the end of the 3rd and 6th weeks. Rats were classified as CSIS if their immobility time was increased by more than 20% from baseline at the end of the 3rd and 6th weeks of testing. CSIS rats treated with Flx were classified as effectively responding to Flx treatment if their immobility behavior decreased by more than 20% at the end of the 6th week compared to CSIS at the end of the 3rd or 6th week. Rats that displayed resilience following 3 weeks of CSIS, i.e., no immobility increase compared to baseline (CSIS resilient), and CSIS + Flx rats which showed no immobility decline at the end of the 6th week (Flx resilient) compared to CSIS rats, were excluded from the current study. The final number of rats per group was 6–8. The experiments were conducted only in male rats since depression induced by social factors in humans has been linked with a higher risk of mortality in males [50], whereby metabolome changes depend on the estrous cycle [51].

### 2.5. Metabolomics Analysis by LCH–RMS

#### 2.5.1. Sample Preparation for LCH–RMS Analysis

Blood samples were collected via cardiac puncture, allowed to clot for 2 h, and centrifuged (at 2000× *g*) for 10 min at room temperature. The serum supernatant was aliquoted and stored at −80 °C until further analysis. Six serum samples each for CSIS and Control, and eight serum samples for Control + Flx and CSIS + Flx, were used for LCH–RMS, with no technical replicates. Thawed aliquots of rat serum (100 µL) were subjected to solvent extraction once each with 400 µL of cold methanol/acetone/acetonitrile (1/1/1, *v*/*v*/*v*), containing 2.5 µM Metabolomics Amino Acid Mix Standard (Cambridge Isotope Laboratories, Andover, MA, USA). Samples were vortexed for 10 s after mixing for 15 min at 4 °C and 1000 rpm (ThermoMixer Eppendorf, Waltham, MA, USA). After incubation for 2 h at −20 °C, and vortexed again for 10 s, samples were centrifuged for 10 min at 14,000 rpm at 4 °C. The collected supernatants were evaporated to dryness using vacuum concentrator (SpeedVac Concentrator, ThermoFisher Scientific, Waltham, MA, USA). After reconstituting the dry extracts in 50 µL of methanol/acetonitrile (1:1), they were vortexed for 15 s and centrifuged at 14,000 rpm for 10 min at 4 °C. Following the transfer of supernatants to LC/MS vials, the LC–HRMS analysis was conducted [52]. The quality controls (QCs) consisted of equal aliquots of each sample. The QCs were diluted before the extraction to achieve relative concentrations of 1, 0.8, 0.5, 0.2 and 0.1. The QCs were extracted in the same manner and at the same time as the samples. After the extraction, the undiluted QC was diluted again to achieve relative concentrations of 0.8, 0.5, 0.2 and 0.1. Both sets of QCs were measured in the beginning and the end of the metabolomics run. The QCs diluted before the extraction were additionally measured after each 10–11 samples, resulting in 4-times technical replicates.

Those replicates were used to check for the stability of the signal (CV%) over the run and the undiluted QCs were additionally used for a QC-based normalization (incorporated in the Compound Discoverer workflow) to normalized for a potential drift in the signal (for each identified metabolite individually). Also, the diluted QCs (before extraction) were used to check for the linearity of the signal. Diluted QCs (after extraction) were used to check for the linearity of the internal standards (labeled amino acids).

An extraction blank (water) was also extracted and measured twice, to check for blank signals and carry-over effects. Metabolites were excluded from further statistical analysis if quality criteria were not met (CV%, linearity of the signal). These measures have been described by Folberth, et al. [52]. All solvents were of LC–MS grade quality and were purchased from Merck (Darmstadt, Germany).

#### 2.5.2. Metabolic Profiles Analyzed by LC–HRMS

LCH–RMS analysis was performed on a Dionex Ultimate 3000 RS LC-system coupled to an Orbitrap mass spectrometer (QExactive, ThermoFisher Scientific, Bremen, Germany) equipped with a heated-electrospray ionization (HESI-II) probe [9]. The chromatographic separation was accomplished on a SeQuant ZIC-HILIC column (150 × 2.1 mm, 5 μm) using water with 5 mM ammonium acetate (Merck, Darmstadt, Germany) as eluent A and acetonitrile (Merck, Darmstadt, Germany)/eluent A (95:5, *v*/*v*) as eluent B. The gradient elution method was set with the following conditions: isocratic step of 100% B for 3 min, 100% B to 60% B in 15 min, held for 5 min, returned to initial conditions in 5 min and held for 5 min. The flow rate was 0.5 mL/min. Data was acquired based on a Full MS/data-dependent MS^2^ (top 10) experiment. Data processing was performed using Compound Discoverer 3.1 (ThermoFisher, San Jose, CA, USA). Metabolite identification was based on the exact mass, retention time, fragmentation spectra and isotopic pattern by 2 independent observers who were blinded to the experimental conditions. Both the online library mzCloud and an in-house library [52] were utilized. The final output data includes the compound name, retention time (RT), exact mass-to-charge (m/z) ratio, and relative peak area. All the datasets used in this study (datatrack_id:4362, study_id:ST002901) were deposited on Metabolomics Workbench repository (www.metabolomicsworkbench.org) [53], available at: https://dev.metabolomicsworkbench.org:22222/data/DRCCMetadata.php?Mode=Study&StudyID=ST002901&Access=XjuE8168, accessed on 31 December 2023.

#### 2.5.3. Metabolite Data Statistics and Analysis

In our investigation, the DMEs were identified through the use of both univariate and multivariate statistical analysis, using MetaboAnalyst 5.0 (http://www.metaboanalyst.ca/). In MetaboAnalyst 5.0 peak areas were normalized by the total sum scaling method followed by a log transformation (base 10) to reduce skewness of the data. Univariate statistical analysis for metabolome data for pair-wise group comparisons was conducted using *t*-test followed by appropriate false-discovery rate (FDR q < 0.05) correction by the Benjamini–Hochberg method to adjust for multiple comparison. Fold change (FC) thresholds of >1.25 and <0.75 were set [54]. Next, multivariate analysis was carried out using pair-wise PLS–DA for supervised class discrimination among groups according to recommended parameters to test how well the known groups can be differentiated based on the metabolite set. To explain the model’s fitness, the values of R2X and R2Y were estimated, while the predictive accuracy of its class model was described by Q2. To identify potentially affected biochemical pathways, identified metabolites were subjected to pathway enrichment analysis in MetaboAnalyst 5.0

### 2.6. Identification of Potential Metabolic Markers

The Biomarker Analysis tool in MetaboAnalyst 5.0 was used to assess ROC curve analysis and AUC evaluation in order to evaluate the performance for each metabolite as a potential metabolic marker [55]. In terms of potential marker capacity, metabolites with an AUC > 0.9 were discussed.

### 2.7. SVML–K-Based Binary Classification

The SVM classifier exhibits the best prediction for each pairwise combination of variables [56,57,58,59]. SVM can also detect relevant metabolites in complex samples, such as blood, where PLS–DA has not achieved this [43,60]. Hence, to identify the most predictive metabolites and to account for possible interactions between the features (which are ignored by the ROC/AUC analysis), we used a greedy forward search procedure by repeatedly training SVM–LK on a growing subset of features as described previously [9]. This method, also known as sequential feature selection, works by iteratively adding the best new feature to the set of previously selected features. The set of selected features is initialized as an empty set. Then, each of the remaining features is used to train a single-feature SVML–K classifier, and the feature that results in the highest predictive accuracy on the CV1 test data is selected. The method continues by choosing the feature which, when combined with the previously selected feature, gives the highest CV1 accuracy. We stop when half of the original number of features are selected. The entire procedure was repeated 10 times with different random seeds, and 3-fold cross-validation was used in the inner loop of the procedure for training the SVM–LK.

### 2.8. RF-Based Binary Classification

RFs are ensembles of decision trees, where the final decision is made through a majority vote [33]. Despite the recent prevalence of deep learning methods for processing data modalities, such as images, audio and text, RFs remain among the most popular and powerful methods for tabular datasets with pre-defined features [61].

As a side-product of training RFs, one can obtain the importance of each feature for the classification task at hand. In the process of constructing the nodes of individual trees, each feature is ranked by the decrease in Gini impurity brought by splitting the data according to the value of that feature [62]. The bigger the impurity decrease, the more valuable the feature is for making the correct classification. The impurity decrease scores of each feature are averaged and these averages are normalized so that the final importance scores of all features sum to one. Since the RF training algorithm selects informative features automatically, we do not employ the greedy forward search procedure used with the SVM–LK. Instead, we train the RF using all available features, and report their computed importance.

We selected the number of trees in the forest and the maximum allowed depth of each tree for each binary classification task by maximizing the Out-Of-Bag (OOB) score, which approximates the validation performance by passing examples only through those trees that had no access to it during training [63]. Then, we used the same 10-times repeated 3-fold cross validation procedure to evaluate the performance of the RF classifiers. We used the scikit-learn Python library for our analysis [64].

### 2.9. Statistical Analysis

Statistica 12 and GraphPad Prism 10 were utilized for statistical analysis and graph display. Three-way repeated measures ANOVA with factor treatment (levels: vehicle and Flx), conditions (levels: control and CSIS), and test as a repeated measure (levels: baseline (weeks 0, 3, and 6), was conducted to analyze the immobility, swimming, and climbing behavior, followed by Duncan’s post-hoc test. Significant differences between the groups are indicated at levels *p* < 0.05, *p* < 0.01, and *p* < 0.001. The number of rats within each group was n = 6–8. Pearson’s analysis was used to correlate the serum metabolite levels with the time of immobility behavior in the FST at the end of the 6th week.

## 3. Results

### 3.1. Behavioral Testing

**Figure 2:**
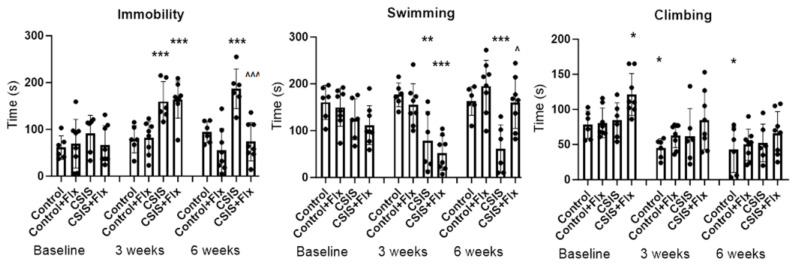
The forced swim test (FST) results in Control (n = 6), fluoxetine-treated control (Control + Flx, n = 8), chronic social isolation (CSIS, n = 6), and effective fluoxetine treatment in CSIS rats (CSIS + Flx, n = 8) at baseline, at the end of the 3rd and 6th week. Differences between groups and controls (baseline) were considered statistically significant at *** *p* < 0.001, ** *p* < 0.01, * *p* < 0.05. Immobility—CSIS or CSIS + Flx (3 weeks) vs. CSIS (baseline), and CSIS (6 weeks) vs. CSIS (baseline) *** *p* < 0.001; CSIS + Flx (6 weeks) vs. CSIS (6 weeks) ^^^^^ *p* < 0.001; Swimming—CSIS (3 weeks) vs. CSIS (baseline) ** *p* < 0.01; CSIS + Flx (3 weeks) vs. CSIS (baseline) and CSIS (6 week) vs. CSIS (baseline) *** *p* < 0.001; CSIS + Flx (6 weeks) vs. CSIS (6 weeks) ^^^ *p* < 0.05; Climbing—CSIS + Flx vs. Control (baseline), * *p* < 0.05; Control (3 weeks) and Control (6 weeks) vs. Control (baseline), * *p* < 0.05. Significant differences between groups were obtained by a three-way repeated measures ANOVA following Duncan’s post-hoc test.

The results of FST are presented in Figure 2. The scoring for immobility, swimming, and climbing behaviors was performed by two independent blinded observers. For immobility time, significant main effects of CSIS (F_1.24_ = 22.21, *p* ˂ 0.001) and Flx (F_1.24_ = 6.42, *p* ˂ 0.05), and effects of time (F_2.48_ = 13.99, *p* ˂ 0.001), CSIS × time (F_2.48_ = 6.74, *p* ˂ 0.01), and Flx × time (F_2.48_ = 10.62, *p* < 0.001) were revealed. At the 3-week and 6-week tests, a significant increase in immobility time in CSIS compared to baseline (*** *p* < 0.001) was revealed. The CSIS + Flx group, prior to treatment with Flx, also showed a significant increase in immobility time at the 3-week test compared to baseline (*** *p* < 0.001). A significant decrease in immobility time in Flx-treated CSIS compared to the CSIS group at the 6-week test (^^^^^ *p* < 0.001) was noted.

For swimming time, a significant main effect of CSIS (F_1.24_ = 26.34, *p* ˂ 0.001), as well as effects of time (F_2.48_ = 5.13, *p* ˂ 0.01), CSIS × time (F_2.48_ = 5.72, *p* ˂ 0.01), and Flx × time (F_2.48_ = 13.38, *p* < 0.001) were observed. At the 3-week and 6-week tests, a significant decrease in swimming time in CSIS compared to baseline (** *p* < 0.01, *** *p* < 0.001, respectively) was found, while 3 weeks of Flx treatment reversed this effect at the end of the 6th week (^^^ *p* < 0.05). Prior to treatment, the CSIS + Flx group displayed a significant decrease in swimming time at the 3-week test compared to baseline (*** *p* < 0.001). Significant main effects of CSIS (F_1.24_ = 5.87, *p* < 0.05) or Flx treatment (F_1.24_ = 4.84, *p* < 0.05), as well as effects of time (F_2.48_ = 19.23, *p* ˂ 0.001) on climbing behavior were observed. A significant increase in climbing time in CSIS + Flx group compared to controls at baseline (* *p* < 0.05) and a significant decrease in controls at the 3-week and 6-week tests compared to baseline (* *p* < 0.05) were found.

### 3.2. Serum Metabolic Profiling Following CSIS with or without Flx Treatment

In the LCH–RMS analysis, 101 annotated metabolites were identified (Supplementary Table S1). The list of DEMs is presented in Table 1.

### 3.3. Multivariate Data Analysis

**Figure 3:**
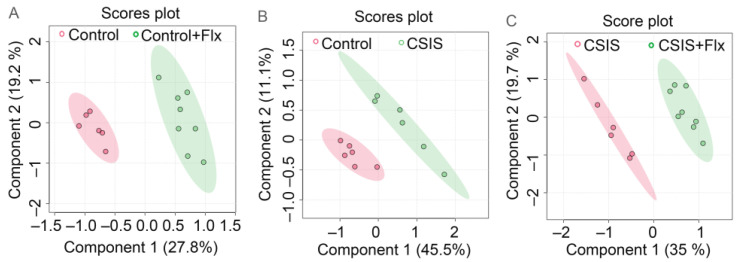
Score plots of PLS – DA show separation among Control + Flx vs. Control (**A**), CSIS vs. Control (**B**), and CSIS + Flx vs. CSIS (**C**). Each dot represents the function of the metabolic profile of an individual sample. The final number of individual measurements of each variable was n = 6–8.

Pair-wise PLS–DA analyses were performed to discriminate groups based on the metabolomic profiles. The key parameters, R^2^ and Q^2^, in pairwise groups were higher than 0.5 (Table 2), indicating that models were robust and had good fitness and prediction. A clear group difference was found among Control + Flx vs. Control (Figure 3A), CSIS vs. Control (Figure 3B) and CSIS + Flx vs. CSIS groups (Figure 3C).

Moreover, Supplementary Figures S1–S3 provide dendrogram (A) and heatmaps (B) of the hierarchical cluster analysis, i.e., metabolite changes for pairwise comparisons.

### 3.4. Identification of Potential Metabolic Markers

For each serum annotated metabolite, molecular marker performance for the CSIS-induced depressive-like behavior and Flx efficacy in CSIS were investigated using the binary logistic regression model, i.e., the classical univariate ROC curve and AUC analysis (Table 3), with the use of the Biomarker Analysis tool within MetaboAnalyst 5.0.

**Figure 4:**
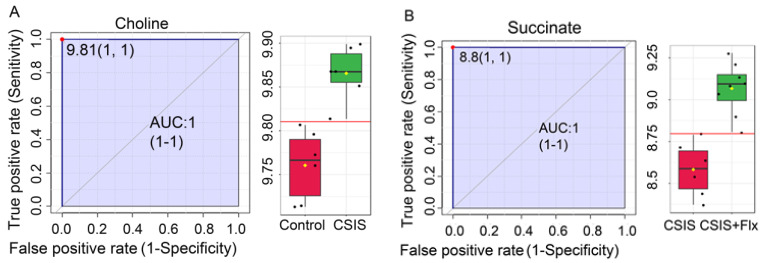
Potential serum markers of CSIS rats that show depressive-like behavior (**A**) and effectively Flx-treated CSIS rats (**B**) based on a classical ROC curve with AUC values. ROC curves are presented with a 95% confidence interval and AUC values. Box-and-whisker plots display individual variable distributions within each group. Red dots (ROC curves) and red lines (box-and-whisker plots) represent the optimal cut-off value between the groups. The final number of individual measurements of each variable was n = 6–8.

According to the ROC analysis, choline with AUC = 1 had the best molecular marker preference for CSIS group designation (Figure 4A), whereas succinate (Figure 4B), along with stachydrine, 7-methylguanine, kynurenic acid, and 5′-methylthioadenosine, had the greatest AUC (1) values, and these were the most significant potential markers following effective Flx treatment in CSIS rats. Moreover, nine metabolites were selected as common potential markers between Control + Flx vs. Control and CSIS + Flx vs. CSIS groups, whereby stachydrine and 7-methylguanine had the best molecular marker preferences among them (Supplementary Table S2).

### 3.5. SVM–LK Classification and Predictive Features

**Figure 5:**
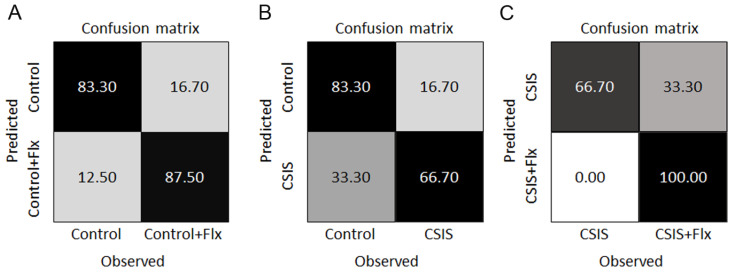
Confusion matrices for SVM–LK for the binary classification of Control + Flx vs. Control (**A**), CSIS vs. Control (**B**) and CSIS + Flx vs. CSIS (**C**). Each of the confusion matrices is visualized as a grey-coded heat map. The final number of individual measurements of each variable was n = 6–8, with no technical replicates.

Performance of the SVM–LK in terms of the confusion matrices for the classification of Control + Flx vs. Control, CSIS vs. Control, and CSIS + Flx vs. CSIS is presented in Figure 5A–C. SVM–LK achieved a panel of seven metabolites for discrimination between CSIS vs. Control rats. In Flx-treated rats (CSIS or Controls), a panel of 9 or 10 metabolites was revealed that could be used for predicting behavioral normalization in CSIS rats by Flx treatment, i.e., Flx effectiveness, as well as behavior phenotype in control rats. The SVM–LK classifier with the most contributing metabolites is presented in Table 4.

### 3.6. RF Classification and Feature Importance

**Figure 6:**
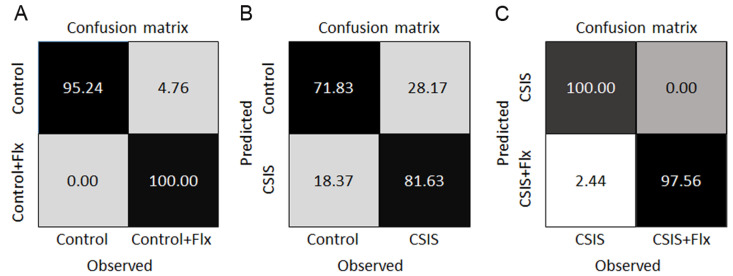
Confusion matrices for RF binary classification of Control + Flx vs. Control (**A**), CSIS vs. Control (**B**) and CSIS + Flx vs. CSIS (**C**). Each of the confusion matrices is visualized as a grey-coded heat map. The final number of individual measurements of each variable was n = 6–8, with no technical replicates.

Performance of the RF in terms of the confusion matrices for the classification of Control + Flx vs. Control, CSIS vs. Control and CSIS + Flx vs. CSIS is presented in Figure 6A–C. Table 5 summarizes the binary classification metrics and the importance of the top 15 metabolites as determined by the RFs. The sensitivity and specificity were computed by considering the first listed class as the positive (Control + Flx, CSIS, and CSIS + Flx, respectively). All metabolites with non-zero importance are presented in Supplementary Table S4.

### 3.7. Correlation of Behavioral Phenotype with Serum Metabolomics

A Pearson correlation analysis was conducted between all metabolites and immobility time at 6th weeks in the FST. The results are shown in Table 6.

**Figure 7:**
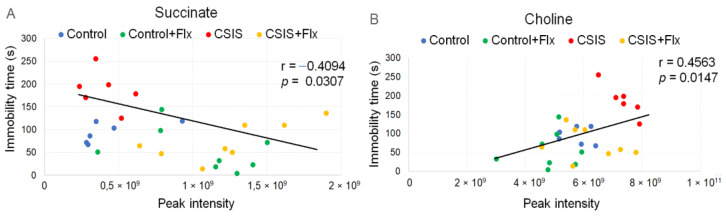
A scatter plot of the metabolite peak intensity of succinate (**A**), choline (**B**), and immobility time in the FST at the end of the 6th week. The black line is the trendline resulting from linear fitting. Six rats each for CSIS and Control groups, and eight for Control + Flx and CSIS + Flx groups, were used for this analysis with no technical replicates.

Pearson’s correlation revealed moderate correlations between the serum metabolite’s levels and immobility time in the FST. Thus, seven metabolites were significantly negatively correlated with immobility time, whereby a medium negative moderate correlation of succinate was found (Figure 7A). In contrast, choline, as a potential marker and predictive metabolite for depressive-like behavior, was positively correlated with the immobility time of FST (Figure 7B). These findings suggest that an altered serum metabolome could reflect depression-like behavior and effective Flx treatment in CSIS rats.

## 4. Discussion

In this study, based on the serum metabolome changes measured by LC–HRMS, we suggest a panel of potential serum markers and ML-driven predictive metabolites for differentiation or discrimination of CSIS vs. Controls and Flx effectiveness in CSIS vs. CSIS rats. Given the possible role of biomarkers in future studies, we will focus on the biological and biochemical interpretation of changed metabolites as potential markers and predictors for depressive phenotype and mode of Flx treatment.

The serum metabolomics profiling of CSIS rats shows significantly altered levels of metabolites that are part of glycerophospholipid, glutamine, and polyamine metabolism. According to ROC/AUC analysis, choline (FC 1.27) was found as the best potential molecular marker and predictive metabolite, according to both classifiers, for CSIS-induced depressive-like behavior. It is an essential precursor in the synthesis of the neurotransmitter acetylcholine [65] and phospholipids, and it plays a role in the facilitation of cholesterol transport in the brain [66,67]. Increased brain choline levels have been associated with an increased risk of depression [68]. Additionally, a significant moderately positive correlation (r = 0.453) between choline and immobility time in the FST was revealed, confirming that it could be used as a potential marker for depressive-like behavior of CSIS rats.

CSIS significantly decreased the contents of N1-acetylspermidine and glutamyl-glutamine, which were also selected as potential markers and predictive metabolites by RF, contributing to the CSIS designation. Reduced N-acetyl-spermidine contents have been linked with affected neurotransmission and cell excitability [69]. Glutamyl–glutamine decline may reflect a deficiency in the glutamyl transferase system (either in its level or activity) that results in a compromised glutamate cycle, which impacts the transport and utilization of amino acids and intracellular glutathione regeneration [70,71]. A tendency toward a reduction was also seen for glutamine (unadjusted *p* value < 0.05, FC 0.72), and it was revealed as a potential marker and predictive metabolite from both classifiers. Glutamine is a precursor of glutamate and gamma-aminobutyric acid (GABA) [72,73], and changing amounts of circulating glutamine may have an impact on the brain’s levels of GABA. CSIS reduced the glycine content (unadjusted *p* value < 0.05, FC 0.87), one of the local inhibitory neurotransmitters and crucial cofactor in N-Methyl-D-aspartate receptor (NMDAR)-mediated signaling, which suggests a potential change in this signaling pathway. Amino acids, such as lysine, histidine, and arginine, with a trend toward a decrease in their levels (unadjusted *p* value < 0.05), were also revealed as potential markers and predictive metabolites according to RF. A recent study demonstrated that dietary arginine decreases oxidative damage and improves brain mitochondrial functioning [74], so its reduced levels in our study may indicate alleviation of serum oxidative stress [75]. Given that amino acid levels are linked to cognitive performance, decreased contents of these metabolites could be related to the cognitive impairments observed in depression [76]. Moreover, identified amino acids were either neurotransmitter precursors (glutamine, arginine), derivatives (methionine, proline), or neurotransmitters (glycine). Changes in the metabolism of amino acids may point to alterations in the synthesis and turnover of neurotransmitters, a process that has been extensively studied in depression [77].

Regarding SVM–LK and RF classifiers, they achieved almost the same accuracy in classifying CSIS vs. Controls (75%, and 75.83% respectively), with a panel of 7 and 42 metabolites, respectively (Table 4 and Supplementary Table S4). The top four potential markers were the most predictive metabolites according to RF, i.e., choline, glutamine, N-acetylspermidine and 5-methylthioadenosine. The other potential metabolite markers (Table 3) were also identified as predictive metabolites according to RF, but with decreased importance (Supplementary Table S4). Despite the fact that other listed predictive metabolites according to SVM–LK were not detected by ROC/AUC analysis or recognized as significantly altered by univariate analysis, their combination with other metabolites may contribute to the CSIS vs. Controls class discrimination [33]. It is therefore possible that metabolite signatures may show specific differences between CSIS and controls, particularly those associated with lipid, amino acid, and polyamine metabolism.

Regarding potential markers for effective Flx treatments in CSIS rats, ROC/AUC analysis selected 13 metabolites, among which succinate, stachydrine, 7-methylguanine, kynurenic acid (KYNA), and 5′-methylthioadenosine were the most significant (Table 3). The increased content of tricarboxylic acid intermediate succinate (FC 3.04) may suggest Flx-driven acceleration of cellular energy metabolism. Additionally, succinate also correlated negatively with immobility behavior (r = −0.4094). An increase in succinate (TCA), 7-methylguanine (guanine containing purine metabolism), 5′-methylthioadenosine (amino acid metabolism), and stachydrine (anti-oxidative mechanism) content, which were moderately negatively correlated with the immobility time in the FST, might be a potential response to effective behavior normalization. In support of this, antidepressant-induced alterations in the metabolism of purines have been noted in both mice and humans [78]. Moreover, in raising the amount of these metabolites, Flx may improve social functioning and cognitive functions. Besides, among others, the levels of prolyl-leucine, aspartate, N-acetyl-glycine, histamine, and choline were positively correlated with immobility time in the FST, whereby their levels may suggest symptoms of behavioral despair in CSIS rats.

Statistically significant altered levels of relevant NMDAR modulators, such as KYNA and histamine, were revealed as potential markers and predictive metabolites for effective Flx treatment in CSIS. It has been suggested that overstimulation of NMDARs due to excessive glutamate release has a role in the pathogenesis of depression [79,80,81], while glutamate release decreases with effective Flx treatment [82]. KYNA is a branch of the kynurenine pathway of tryptophan metabolism [83] and an antagonist at the glycine site of the NMDAR complex [84] that reduces glutamatergic transmission. It also regulates oxidative and nitrosative stress pathways and reduces the production of inflammatory factors [85]. Contrary to this, histamine acts as a positive allosteric modulator of the NMDAR [86]. Therefore, opposing metabolic levels of KYNA (FC 2.29) and histamine (FC 0.51) could represent the changes that cells make to regulate NMDAR stimulation following effective Flx treatment in CSIS rats.

Potential markers such as succinate, stachydrine, 7-methylguanine, and 5′-methylthioadenosine, with the best molecular preference were also detected as predictive metabolites for Flx efficacy in CSIS with high importance via RF. When comparing Flx-induced metabolic changes in the CSIS rats with those observed in Flx-treated controls, nine matched potential markers were found (Supplementary Table S2). Notably, Flx-induced metabolic changes in control rats were not associated with alterations in immobility behavior in the FST, suggesting adaptive cellular responses to chronic Flx treatment or behavior independent effects. SVM–LK showed five predictive metabolites with a similar pattern of action in both Flx-treated groups (Control or CSIS) (Supplementary Table S3). In addition, three matched predictive metabolites, amino(iso)butyric acid, urea, and choline, in CSIS group were also shown to be powerful in designating Flx efficacy in CSIS rats compared to CSIS (Supplementary Table S3). We may assume that altered homeostasis of cells exposed to CSIS may lead to different susceptibilities of cells to Flx treatment.

The RF classifier showed better predictive accuracy than SVM LK (98.50% vs. 85.70%, respectively) in designation of Flx efficacy in CSIS (Supplementary Table S5). In addition, carnitines, such as, butanol-carnitine, acetyl-l-carnitine, palmitoyl-carnitine, hexanoyl-carnitine, deoxy-carnitine, and propionyl-carnitine (unadjusted *p* value < 0.05, FC 1.04–1.66), were detected as predictive metabolites for Flx efficacy in the CSIS group. However, it remains to be explored if the enhancement of these metabolites represents a potential target for Flx efficacy or, more probably, reflects an efficient fueling of carbons from fatty acids to the TCA cycle. Currently, we cannot explain every individual finding, and we can only speculate about the relevant biochemical pathways. Moreover, the use of RF is optimal for dealing with so called “large *p*, small *n*” data sets [87] by minimizing the potential for overfitting data. A limitation of the current study is that each animal group consisted of six or eight animals. However, follow-up work with a larger cohort is required to independently validate our findings.

## 5. Conclusions

In the present study, the rat serum metabolic changes of CSIS rats and the effectiveness of Flx treatment in CSIS were investigated by LC–HRMS associated with identifying potential markers and predictive metabolites using ROC/AUC analysis and ML algorithms. The significantly changed metabolome induced by CSIS revealed disturbances in amino acid metabolism, lipid metabolism, and polyamine metabolism. The increased serum choline was revealed as a potential marker for CSIS-induced depressive-like behavior. A panel of 7 or 42 serum metabolites, obtained by SVM–LK or RF, could be used as predictors for CSIS vs. Controls. Among others, the anti-oxidative mechanism (stachydrine), guanine containing purine metabolism (7-methylguanine), amino acid metabolism (5′-methylthioadenosine), mitochondrial energy metabolism (succinate), and modulation of glutamatergic neurotransmission on NMDAR (KYNA) might aid in recovery from depressed-like behavior. RF showed better accuracy than SVM LK in discriminating Flx efficacy in CSIS vs. CSIS with a panel of 51 predictive metabolites, indicative of behavioral normalization in rats. Although we used a rat model of depression, these results indicate that it is promising to search diagnostic, predictive, and therapeutic biomarkers combining mass spectrometry-based untargeted metabolomics and ML methods through a non-invasive serum sample. Additionally, this study established a basis for future research on brain organoids and human serum samples.

## Figures and Tables

**Table 1:** List of DEMs following chronic social isolation (CSIS) as compared to Control, Flx-treated controls as compared to Control, and effective Flx treatment in CSIS rats as compared to CSIS, detected by LCHR–MS. False discovery rate (FDR)-adjusted *p*-values (*t*-test) < 0.05 and fold change (FC) > 1.25 and <0.75 were criteria for DEMs. RT, retention time.

		Control + Flx vs. Control			CSIS vs. Control			CSIS + Flx vs. CSIS			
RT (min)	Metabolite	*p*-Value	p-Adjusted	FC	*p*-Value	p-Adjusted	FC	*p*-Value	p-Adjusted	FC	Pathway
3.57	Hydroxy-hippuric acid	2.02 × 10^−3^	2.55 × 10^−3^	0.34				5.87 × 10^−4^	6.60 × 10^−3^	0.38	Lipid metabolism
19.73	Histamine	6.13 × 10^−4^	1.24 × 10^−2^	0.38				2.00 × 10^−4^	2.90 × 10^−3^	0.51	Amino acid metabolism
1.33	Hydroxy-hexa-decanoic acid	6.83 × 10^−5^	2.15 × 10^−3^	0.45							Lipid metabolism
2.02	Uracil	3.94 × 10^−3^	3.62 × 10^−2^	0.54							Nucleotide metabolism
12.35	Aspartate	1.94 × 10^−3^	2.55 × 10^−2^	0.59				2.84 × 10^−4^	3.60 × 10^−3^	0.49	Amino acid metabolism
2.41	3-(4-Hydroxy-phenyl) lactate	8.91 × 10^−4^	1.50 × 10^−2^	0.64							Energy metabolism
5.35	Riboflavin	5.41 × 10^−3^	4.56 × 10^−2^	1.39							Riboflavin metabolism
8.84	Deoxy-carnitine	6.29 × 10^−3^	4.89 × 10^−2^	1.40							Lipid metabolism
6.63	1-Methylxanthine	2.72 × 10^−3^	3.05 × 10^−2^	1.87				1.54 × 10^−4^	2.60 × 10^−3^	2.66	Purine metabolism
6.63	7-Methylguanine	8.50 × 10^−5^	2.15 × 10^−3^	1.88				6.00 × 10^−7^	6.06 × 10^−5^	2.63	Purine metabolism
7.95	Propionyl carnitine	1.72 × 10^−5^	8.71 × 10^−4^	2.04				1.95 × 10^−3^	1.78 × 10^−2^	1.66	Amino acid metabolism
2.04	Succinate	3.65 × 10^−3^	3.62 × 10^−2^	2.42				9.87 × 10^−5^	2.0 × 10^−3^	3.04	Energy metabolism
9.98	Stachydrine	2.20 × 10^−6^	2.00 × 10^−4^	3.27				1.09 × 10^−5^	5.51 × 10^−4^	2.30	Energy metabolism
19.20	N1-Acetyl spermidine				2.78 × 10^−4^	2.36 × 10^−2^	0.31				Polyamine metabolism
13.04	Glutamyl-glutamine				1.41 × 10^−3^	4.76 × 10^−2^	0.34				Amino acid metabolism
8.48	Choline				4.68 × 10^−4^	2.36 × 10^−2^	1.27				Lipid metabolism
2.22	N-Acetyl-glycine							2.44 × 10^−3^	2.06 × 10^−2^	0.69	Amino acid metabolism
11.72	Guanidino acetate							1.49 × 10^−3^	1.49 × 10^−2^	1.86	Amino acid metabolism
6.04	Kynurenic acid							2.11 × 10^−5^	7.10 × 10^−4^	2.29	Amino acid metabolism
2.15	5′-Methylthio adenosine							4.98 × 10^−5^	1.26 × 10^−2^	2.71	Amino acid metabolism

**Table 2:** PLS–DA classifier performances.

Group Comparison	No of Components	R^2 a^	Q^2 b^	Accuracy
Control + Flx vs. Control	5	0.99987	0.80464	1
CSIS vs. Control	5	0.99991	0.69004	0.93
CSIS + Flx vs. CSIS	5	0.99989	0.93421	1

^a^ Measure of goodness of fit of the model; ^b^ Measure of predictive ability of the model.

**Table 3:** List of serum metabolites as potential markers for differentiating Flx treatments in Control vs. Control, CSIS vs. Control, and effective Flx treatments in CSIS vs. CSIS that had an AUC > 0.90.

Control + Flx vs. Control			CSIS vs. Control			CSIS + Flx vs. CSIS		
Metabolites	AUC	FC	Metabolites	AUC	FC	Metabolites	AUC	FC
Stachydrine	1	3.27	Choline	1	1.27	Succinate	1	3.04
7-Methylguanine	1	1.88	Glutamine	1	0.72	Stachydrine	1	2.30
3-(4 Hydroxy phenyl) lactate	1	0.64	N1-Acetyl spermidine	1	0.31	7-Methylguanine	1	2.63
Propionyl-carnitine	1	2.04	Glutamyl-glutamine	0.97222	0.34	Kynurenic acid	1	2.29
Hydroxy-hexa-decanoic acid	1	0.45	5′-Methylthio- adenosine	0.97222	0.58	5′-Methylthio adenosine	1	2.71
Histamine	0.97917	0.38	Lysine	0.94444	0.63	Histamine	0.97917	0.51
1-Methyl- xanthine	0.95833	1.87	Histidine	0.94444	0.75	Aspartate	0.97917	0.49
Hydroxy hippuric acid	0.95833	0.34	Urate	0.91667	0.67	1-Methyl xanthine	0.97917	2.66
Uracil	0.91667	0.54	Arginine	0.91667	0.72	Guanidino acetate	0.95833	1.86
Succinate	0.91667	2.42	Cystathionine	0.91667	0.52	Hydroxy hippuric acid	0.95833	0.38
Aspartate	0.91667	0.59				Propionyl carnitine	0.95833	1.66
Deoxy-carnitine	0.91667	1.40				N-Acetyl-glycine	0.9375	0.69
3-Hydroxy-3-methylglutarate	0.91667	0.66				Hydroxy-hexa-decanoic acid	0.91667	0.75
Urocanate	0.89583	0.60						

**Table 4:** SVM–LK-based binary classification performance for pair-wise comparisons of the rat serum metabolite samples, FC-fold change.

Control + Flx vs. Control		CSIS vs. Control		CSIS + Flx vs. CSIS	
Accuracy	85.70%	Accuracy	75.00%	Accuracy	85.70%
Sensitivity	83.30%	Sensitivity	83.30%	Sensitivity	66.70%
Specificity	87.50%	Specificity	66.70%	Specificity	100.00%
Balanced Accuracy	85.40%	Balanced Accuracy	75.00%	Balanced Accuracy	85.30%
Predictive Metabolites	FC	Predictive Metabolites	FC	Predictive Metabolites	FC
Histamine	0.38	Glutamine	0.72	Histamine	0.51
Uracil	0.54	Lactate	0.79	N-Acetyl-glycine	0.69
Aspartate	0.59	Amino(iso)butyric acid	0.82	Choline	0.84
Putrescine	0.81	Glycine	0.87	Cytosine	1.14
N-Acetyl-glycine	0.82	Indole	0.88	Urea	1.14
Pyruvate	0.82	Urea	0.96	Amino(iso)butyric acid	1.31
Choline	0.84	Choline	1.27	Guanidinoacetate	1.86
Urea	1.12			Stachydrine	2.30
Succinate	2.42			Succinate	3.04
Stachydrine	3.27				

**Table 5:** RF-based binary classification performance for pair-wise comparisons and the importance of the top 15 metabolites, FC-fold change.

Control + Flx (Positive) vs. Control			CSIS (Positive) vs. Control			CSIS + Flx (Positive) vs. CSIS		
Accuracy	98.00%		Accuracy	75.83%		Accuracy	98.50%	
Sensitivity	96.67%		Sensitivity	66.67%		Sensitivity	100.00%	
Specificity	100.00%		Specificity	85.00%		Specificity	96.67%	
Balanced Accuracy	98.33%		Balanced Accuracy	75.83%		Balanced Accuracy	98.33%	
Predictive metabolites			Predictive metabolites			Predictive metabolites		
Name	Import ance	FC	Name	Import ance	FC	Name	Import ance	FC
Hydroxy-hexa -decanoic acid	0.1354	0.45	5′-Methylthio adenosine	0.1327	0.58	Stachydrine	0.0767	2.30
Histamine	0.0807	0.38	Choline	0.1200	1.27	7-Methyl guanine	0.0767	2.63
Stachydrine	0.0800	3.27	N1-Acetyl spermidine	0.0714	0.31	5′-Methyl thio-adenosine	0.0706	2.71
7-Methylguanine	0.0658	1.88	Myo-Inositol	0.0633	0.71	1-Methyl xanthine	0.0662	2.66
1-Methyl xanthine	0.0574	1.87	Glutamine	0.0429	0.72	Kynurenic acid	0.0600	2.29
Propionyl carnitine	0.0553	2.04	Lysine	0.0429	0.63	Succinate	0.0600	3.04
3-(4-Hydroxy phenyl) lactate	0.0458	0.64	Cystathionine	0.0386	0.52	Aspartate	0.0554	0.49
Aspartate	0.0413	0.59	Arginine	0.0343	0.72	Guanidino acetate	0.0412	1.86
Hydroxy hippuric acid	0.0356	0.34	Allantoin	0.0340	0.85	Glycero-phospho-choline	0.0317	0.74
Riboflavin	0.0274	1.39	N-Acetyl cytidine	0.0302	0.82	Choline	0.0292	0.84
Ala-Pro (Alanyl-Proline)	0.0244	1.65	Methionine	0.0288	0.82	Histamine	0.0281	0.51
Anserine	0.0228	0.59	Indole	0.0286	0.88	N-Acetyl glycine	0.0219	0.69
Succinate	0.0228	2.42	Hydroxy-hexa -decanoic acid	0.0257	0.78	Hydroxy-hexa -decanoic acid	0.0217	0.75
Urocanate	0.0200	0.60	Asparagine	0.0213	0.78	N1-Acetyl spermidine	0.0200	3.67
Orotic acid	0.0200	0.65	Vanillic acid	0.0143	0.74	Propionyl carnitine	0.0200	1.66

**Table 6:** Statistically significant Pearson correlation (0.4 < r < −0.4), *p* < 0.05, between the serum metabolites and immobility behavior.

Metabolites Content vs. Immobility Pearson Correlation	r	*p*
N1-Acetylspermidine	−0.5274	0.0040
Stachydrine	−0.4934	0.0077
7-Methylguanine	−0.4841	0.0091
5′-Methylthioadenosine	−0.4738	0.0110
Glutamyl-leucine	−0.4099	0.0307
Succinate	−0.4094	0.0307
Glutamyl-glutamine	−0.4045	0.0330
Hydroxy-hippuric acid	0.4349	0.0207
Choline	0.4563	0.0147
Anserine	0.4657	0.0125
Histamine	0.4765	0.0104
3-Hydroxy-3-methylglutarate	0.5166	0.0049
N-Acetyl-glycine	0.5324	0.0035
Aspartate	0.5493	0.0025
Prolyl-leucine	0.5627	0.0018

## Data Availability

Metabolomics data are available in https://dev.metabolomicsworkbench.org:22222/data/DRCCMetadata.php?Mode=Study&StudyID=ST002901&Access=XjuE8168.

## Supplementary Materials

The following supporting information can be downloaded at: https://www.mdpi.com/article/10.3390/metabo14080405/s1, Figure S1:Hierarchical clustering dendrogram and heatmaps of Control + Flx vs. Control; Figure S2: Hierarchical clustering dendrogram and heatmaps of CSIS vs. Control; Figure S3: Hierarchical clustering dendrogram and heatmaps of CSIS + Flx vs. CSIS; Table S1: List of annotated rat serum metabolites found in each sample; Table S2: List of overlapped rat serum metabolites between CSIS + Flx vs. CSIS, and CSIS vs. Control, and Control + Flx vs. Control that had an AUC > 0.90; Table S3: List of overlapped rat serum predictive metabolites from SVM-LK for classification CSIS + Flx vs. CSIS, CSIS vs. Control and Control + Flx vs. Control; Table S4: List of metabolites for the classification of Control + Flx vs. Control, CSIS vs. Control and CSIS + Flx vs. CSIS obtained by Random Forest; Table S5: List of overlapped rat serum predictive metabolites from RF for classification CSIS + Flx vs. CSIS, CSIS vs. Control and Control + Flx vs. Control.
