# Supplementary material for: Serum Metabolites as Potential Markers and Predictors of Depression-like Behavior and Effective Fluoxetine Treatment in Chronically Socially Isolated Rats
Source: Metabolites. 2024 Jul 25;14(8):405. doi: 10.3390/metabo14080405 (PMC11355999; doi:10.3390/metabo14080405)

Supplementary Figure S1

A Hierarchical clustering dendrogram of Control + Flx vs. Control

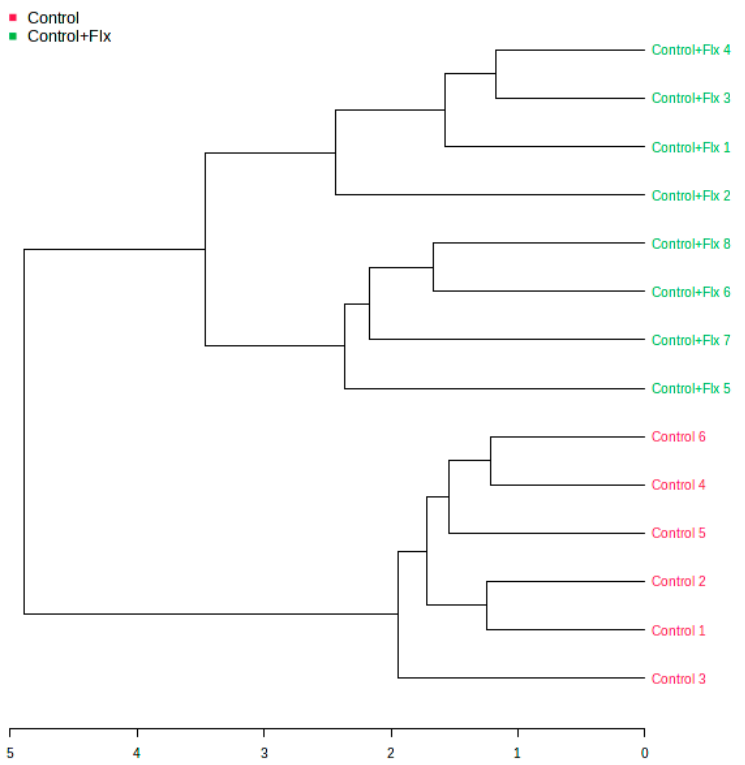

B Hierarchical clustering heatmaps Control + Flx vs. Control

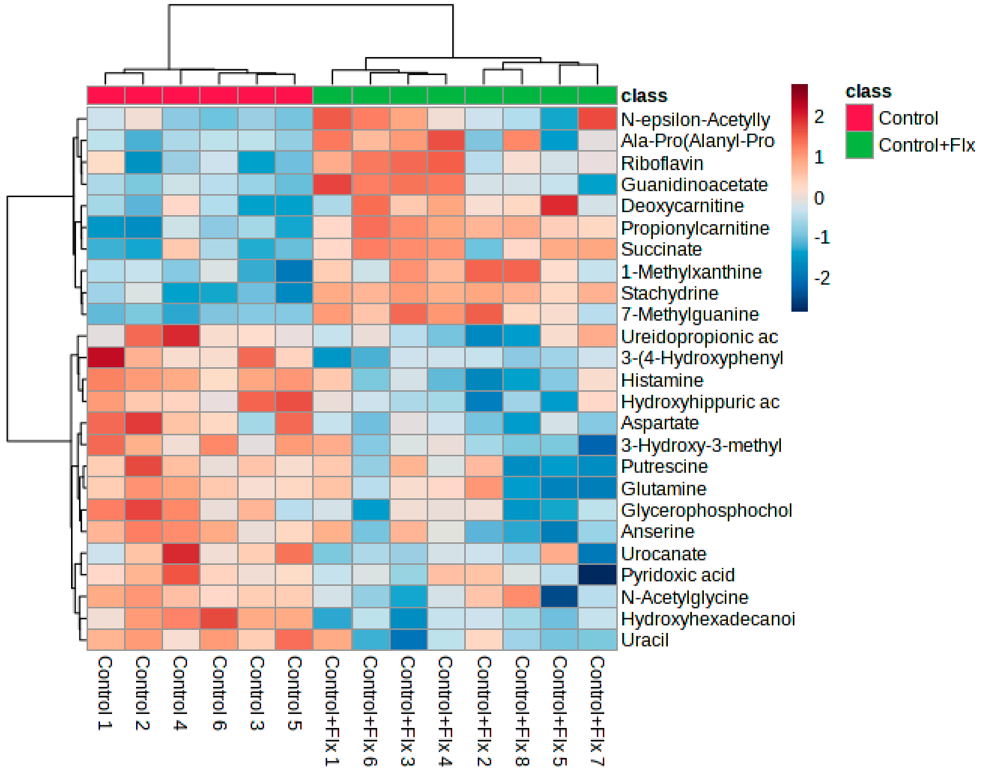

Supplementary Figure S2

A Hierarchical clustering dendrogram of CSIS vs. Control

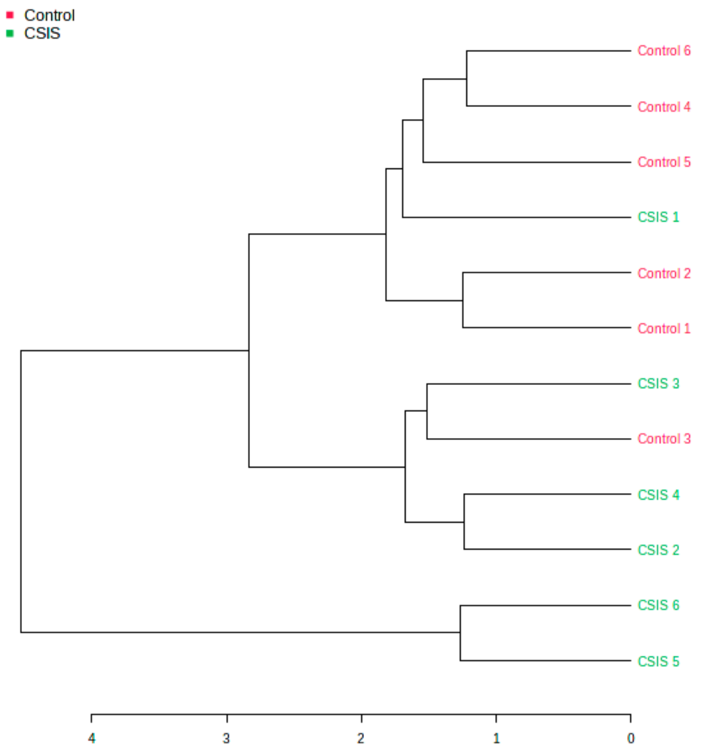

B Hierarchical clustering heatmaps CSIS vs. Control

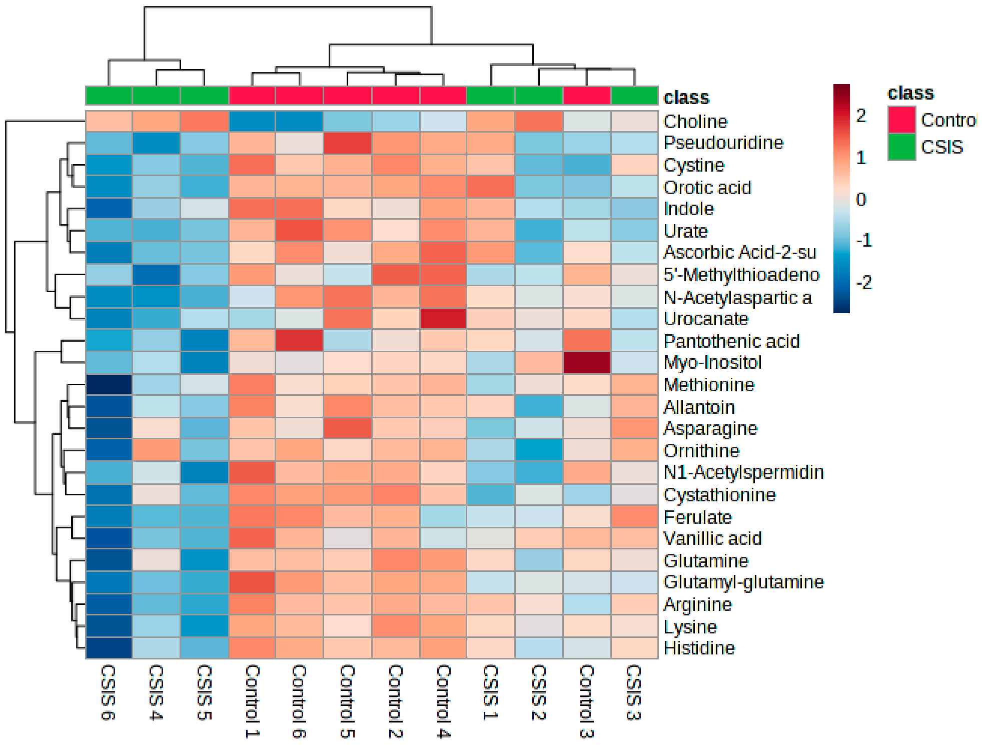

Supplementary Figure S3

A Hierarchical clustering dendrogram of CSIS + Flx vs. CSIS

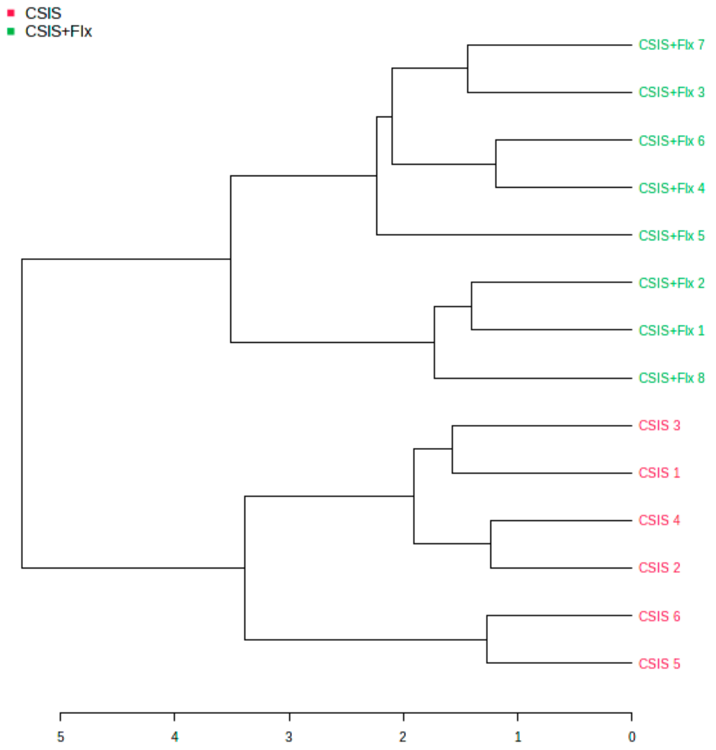

B Hierarchical clustering heatmaps CSIS + Flx vs. CSIS

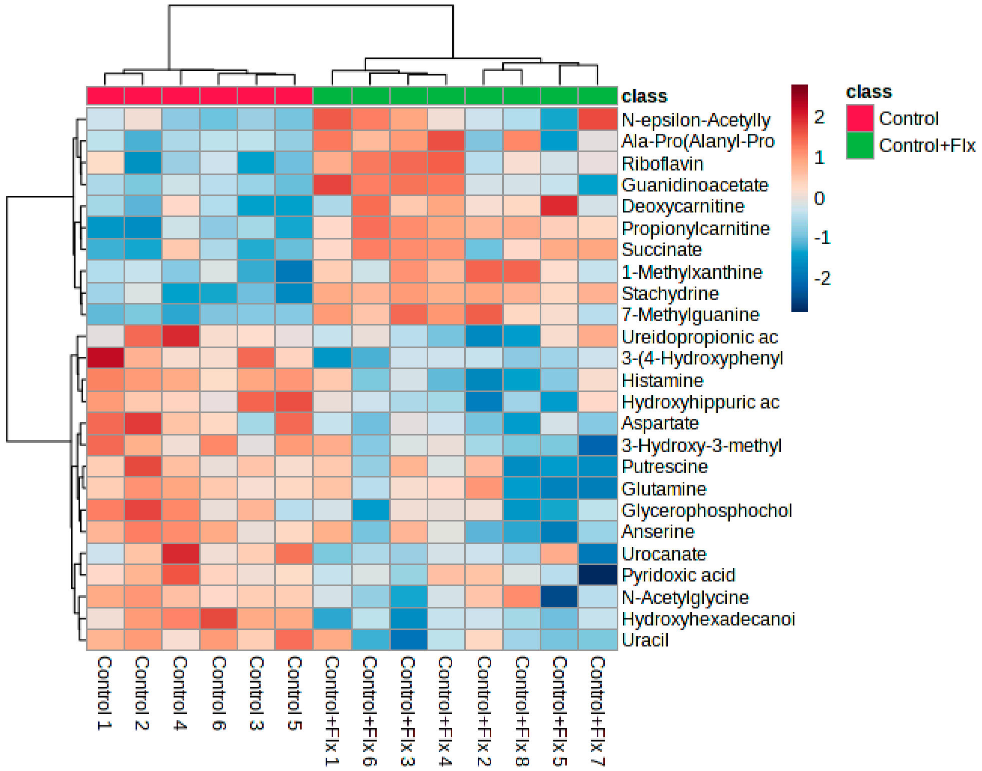

Supplement: Supplementary file 1 [file metabolites-14-00405-s001.zip › Supplementary Figures S1-S3.pdf]
