# Supplementary material for: Serum Metabolites as Potential Markers and Predictors of Depression-like Behavior and Effective Fluoxetine Treatment in Chronically Socially Isolated Rats
Source: Metabolites. 2024 Jul 25;14(8):405. doi: 10.3390/metabo14080405 (PMC11355999; doi:10.3390/metabo14080405)
Supplement: Supplementary file 1 [file metabolites-14-00405-s001.zip › Supplementary Tables S2-S5.pdf]

Supplementary Table 2. List of overlapped rat serum metabolites between CSIS+Flx vs. CSIS, and CSIS vs. Control, and Control+Flx vs. Control that had an AUC > 0.90

	**CSIS+Flx vs. CSIS**		**CSIS vs. Control**		**Control+Flx vs. Control**	
**Metabolites**	AUC	FC	AUC	FC	AUC	FC
5'-Methylthio adenosine	1	2.71	0.97222	0.58		
7-Methylguanine	1	2.63			1	1.88
Stachydrine	1	2.30			1	3.27
Succinate	1	3.04			0.91667	2.42
1-Methylxanthine	0.97917	2.66			0.95833	1.87
Aspartate	0.97917	0.49			0.91667	0.59
Histamine	0.97917	0.51			0.97917	0.38
Hydroxyhippuric acid	0.95833	0.38			0.95833	0.34
Propionylcarnitine	0.95833	1.66			1	2.04
Hydroxyhexa decanoic acid	0.91667	0.75			1	0.45

Supplementary Table 3. List of overlapped rat serum predictive metabolites from SVM-LK for classification CSIS+Flx vs. CSIS, CSIS vs. Control and Control+Flx vs. Control

	**CSIS+Flx vs. CSIS**	**CSIS vs. Control**	**Control+Flx vs. Control**
**Predictive metabolites**	FC	FC	FC
Succinate	3.04		2.42
Stachydrine	2.30		3.27
Amino(iso)butyric acid	1.31	0.82	
Urea	1.14	0.96	1.12
Choline	0.84	1.27	0.84
Histamine	0.51		0.38

Supplementary Table 4. List of metabolites for the classification of Control+Flx vs. Control, CSIS vs. Control and CSIS+Flx vs. CSIS obtained by Random Forest

**Control+Flx (positive) vs. Control**		**CSIS (positive) vs. Control**		**CSIS+Flx (positive) vs. CSIS**	
Accuracy	98.00%	Accuracy	75.83%	Accuracy	98.50%
Sensitivity	96.67%	Sensitivity	66.67%	Sensitivity	100.00%
Specificity	100.00%	Specificity	85.00%	Specificity	96.67%

Balanced Accuracy	98.33%		Balanced Accuracy	75.83%		Balanced Accuracy	98.33%	
**Predictive metabolites**			**Predictive metabolites**			**Predictive metabolites**		
Name	Importance	FC	Name	Importance	FC	Name	Importance	FC
Hydroxyhexadecanoic acid	0.1354	0.45	5'-Methylthioadenosine	0.1327	0.58	Stachydrine	0.0767	2.30
Histamine	0.0807	0.38	Choline	0.1200	1.27	7-Methylguanine	0.0767	2.63
Stachydrine	0.0800	3.27	N1-Acetylspermidine	0.0714	0.31	5'-Methylthioadenosine	0.0706	2.71
7-Methylguanine	0.0658	1.88	Myo-Inositol	0.0633	0.71	1-Methylxanthine	0.0662	2.66
1-Methylxanthine	0.0574	1.87	Glutamine	0.0429	0.72	Kynurenic acid	0.0600	2.29
Propionylcarnitine	0.0553	2.04	Lysine	0.0429	0.63	Succinate	0.0600	3.04
3-(4-Hydroxyphenyl)lactate	0.0458	0.64	Cystathionine	0.0386	0.52	Aspartate	0.0554	0.49
Aspartate	0.0413	0.59	Arginine	0.0343	0.72	Guanidinoacetate	0.0412	1.86
Hydroxyhippuric acid	0.0356	0.34	Allantoin	0.0340	0.85	Glycerophosphocholine	0.0317	0.74
Riboflavin	0.0274	1.39	N-Acetylcytidine	0.0302	0.82	Choline	0.0292	0.84
Ala-Pro (Alanyl-Proline)	0.0244	1.65	Methionine	0.0288	0.82	Histamine	0.0281	0.51
Anserine	0.0228	0.59	Indole	0.0286	0.88	N-Acetylglycine	0.0219	0.69
Succinate	0.0228	2.42	Hydroxyhexadecanoic acid	0.0257	0.78	Hydroxyhexadecanoic acid	0.0217	0.75
Urocanate	0.0200	0.60	Asparagine	0.0213	0.78	N1-Acetylspermidine	0.0200	3.67
Orotic acid	0.0200	0.65	Vanillic acid	0.0143	0.74	Propionylcarnitine	0.0200	1.66
Choline	0.0131	0.85	Cytidine	0.0143	0.89	Hydroxyhippuric acid	0.0183	0.38
Sphingomyelin (d34:1)	0.0126	0.94	Urate	0.0143	0.67	Tauro(cheno)deoxycholic Acid	0.0183	1.57
3-Methylhistidine	0.0101	0.86	Pyridoxic acid	0.0143	0.81	Amino(iso)butyric acid	0.0163	1.31
1-Methylhistidine	0.0100	1.25	Dimethylarginine	0.0143	0.78	Trimethyllysine	0.0152	1.29
Guanidinoacetate	0.0100	1.55	Pantothenic acid	0.0143	0.72	Cytosine	0.0152	1.14
Glutamine	0.8100	0.81	Histidine	0.0143	0.75	Indoxyl sulfate	0.0150	1.53
Kynurenic acid	0.0100	1.63	N-Acetylaspartic acid	0.0143	0.70	Urea	0.0106	1.14
Sphingomyelin (d36:1)	0.0100	0.85	Sphingosine (d18:1)	0.0143	1.43	Allantoin	0.0096	1.28

N-Acetylglycine	0.0100	0.82	Methylcytidine	0.0143	0.88	Palmitoyl carnitine	0.0088	1.34
Protoporphyrin	0.0100	0.85	Histamine	0.0143	0.89	Prolylleucine	0.0086	0.71
Proline	0.0100	1.00	Pseudouridine	0.0143	0.77	N-Methyl histamine	0.0085	1.46
Ascorbic Acid-2-sulfate	0.0100	0.81	Stachydrine	0.0127	1.40	Glutamyl-glutamine	0.0077	1.70
Glutamyl-glutamine	0.0100	0.85	Guanidino acetate	0.0102	0.87	Hexanoyl carnitine	0.0067	1.38
Uracil	0.0100	0.54	Citrulline	0.0100	0.76	PLK	0.0067	1.10
N-Acetylserine	0.0097	0.87	Ascorbic Acid-2-sulfate	0.0100	0.73	Deoxy carnitine	0.0067	1.49
Dimethyl arginine	0.0075	0.98	Carnitine	0.0086	0.82	N-Acetyl cytidine	0.0067	1.42
3-Hydroxy-3-methylglutarate	0.0075	0.66	Cytosine	0.0071	0.97	Sphingomyelin (d34:1)	0.0067	0.90
Lactate	0.0075	0.76	Glutamyl-glutamine	0.0071	0.34	Ureido propionic acid	0.0067	1.10
Ureidopropionic acid	0.0074	0.67	Amino(iso)butyric acid	0.0071	0.82	Cystathionine	0.0067	1.50
Acetyl-L-carnitine	0.0072	0.78	Aspartate	0.0048	1.16	Ascorbic Acid-2-sulfate	0.0067	1.02
Glycerophosphocholine	0.0072	0.67	Acetyl-L-carnitine	0.0043	0.95	Creatinine	0.0067	1.10
Cystathionine	0.0067	0.95	Glycerophosphocholine	0.0043	0.99	3-Methyl histidine	0.0065	1.26
Glutamylleucine	0.0067	1.15	N8-Acetyl spermidine	0.0043	0.89	Cytidine	0.0056	1.05
Amino(iso)butyric acid	0.0054	0.96	Creatinine	0.0041	1.03	Xanthosine	0.0054	1.01
Adenosine	0.0046	0.75	Homocitrulline	0.0041	1.03	Acetyl-L-carnitine	0.0048	1.21
Carnosine	0.0044	0.91	Prolylleucine	0.0041	1.17	Lactate	0.0045	1.07
Myo-Inositol	0.0042	1.25	Phenylalanine	0.0041	0.89	Glycine	0.0041	1.12
Betaine	0.0032	0.98				Methionine sulfoxide	0.0036	0.81
Carnitine	0.0028	0.81				Riboflavin	0.0035	1.43
Pyruvate	0.0026	0.82				Methylcytidine	0.0033	1.18
Trimethyllysine	0.0025	1.10				Citrulline	0.0031	0.92
Ornithine	0.0025	0.89				Butanoyl carnitine	0.0031	1.04
						Sphingomyelin (d36:1)	0.0026	0.89
						Uridine	0.0019	1.21
						Urate	0.0017	1.37
						Pseudouridine	0.0017	1.34

Supplementary Table 5. List of overlapped rat serum predictive metabolites from RF for classification CSIS+Flx vs. CSIS, CSIS vs. Control and Control+Flx vs. Control

	**CSIS+Flx vs. CSIS**		**CSIS vs. Control**		**Control+Flx vs. Control**	
**Predictive metabolites**	Importance	FC	Importance	FC	Importance	FC
Stachydrine	0.0767	2.30	0.0127	1.40	0.0800	3.27
7-Methylguanine	0.0767	2.63			0.0658	1.88
5'-Methylthioadenosine	0.0706	2.71	0.1327	0.58		
1-Methylxanthine	0.0662	2.66			0.0574	1.87
Kynurenic acid	0.0600	2.29			0.0100	1.63
Succinate	0.0600	3.04			0.0228	2.42
Aspartate	0.0554	0.49	0.0048	1.16	0.0413	0.59
Guanidinoacetate	0.0412	1.86	0.0102	0.87	0.0100	1.55
Glycerophosphocholine	0.0317	0.74	0.0043	0.99	0.0072	0.67
Choline	0.0292	0.84	0.1200	1.27	0.0131	0.85
Histamine	0.0281	0.51	0.0143	0.89	0.0807	0.38
N-Acetylglycine	0.0219	0.69			0.0100	0.82
Hydroxyhexadecanoic acid	0.0217	0.75	0.0257	0.78	0.1354	0.45
N1-Acetylspermidine	0.0200	3.67	0.0714	0.31		
Propionylcarnitine	0.0200	1.66			0.0553	2.04
Hydroxyhippuric acid	0.0183	0.38			0.0356	0.34
Amino(iso)butyric acid	0.0163	1.31	0.0071	0.82	0.0054	0.96
Trimethyllysine	0.0152	1.29			0.0025	1.10
Cytosine	0.0152	1.14	0.0071	0.97		
Allantoin	0.0096	1.28	0.0340	0.85		
Prolylleucine	0.0086	0.71	0.0041	1.17		
Glutamyl-glutamine	0.0077	1.70	0.0071	0.34	0.0100	0.85
Cystathionine	0.0067	1.50	0.0386	0.52	0.0067	0.95
Ascorbic Acid-2-sulfate	0.0067	1.02	0.0100	0.73	0.0100	0.81
N-Acetylcytidine	0.0067	1.42	0.0302	0.82		
Creatinine	0.0067	1.10	0.0041	1.03		
Sphingomyelin (d34:1)	0.0067	0.90			0.0126	0.94
Ureidopropionic acid	0.0067	1.10			0.0074	0.67
3-Methylhistidine	0.0065	1.26			0.0101	0.86
Cytidine	0.0056	1.05	0.0143	0.89		
Acetyl-L-carnitine	0.0048	1.21	0.0043	0.95	0.0072	0.78
Lactate	0.0045	1.07			0.0075	0.76
Riboflavin	0.0035	1.43			0.0274	1.39
Methylcytidine	0.0033	1.18	0.0143	0.88		
Citrulline	0.0031	0.92	0.0100	0.76		
Sphingomyelin (d36:1)	0.0026	0.98			0.0100	0.85
Urate	0.0017	1.37	0.0143	0.67		

Pseudouridine	0.0017	1.34	0.0143	0.77		
Myo-Inositol			0.0633	0.71	0.0042	1.25
Glutamine			0.0429	0.72	0.0100	0.81
Dimethylarginine			0.0143	0.78	0.0075	0.98
Carnitine			0.0086	0.82	0.0028	0.81
